# Efficacy of follitropin delta in potential low responders: a pooled analysis of individual patient data from five randomized controlled trials

**DOI:** 10.3389/fendo.2026.1880515

**Published:** 2026-09-04

**Authors:** Rita Lobo, Ali Falahati, Ida E. Jepsen, Nikolaos P. Polyzos, Juan A. García-Velasco, Anja Pinborg, Enrica Gravotta

**Affiliations:** 1 Office of Chief Medical Officer, Ferring Pharmaceuticals, Copenhagen, Denmark; 2 Global Biometrics, Ferring Pharmaceuticals, Copenhagen, Denmark; 3 Dexeus Fertility, Department of Obstetrics, Gynaecology and Reproductive Medicine, Hospital Universitari Dexeus, Barcelona, Spain; 4 IVIRMA Global Research Alliance, IVI Madrid, Madrid, Spain; 5 Rey Juan Carlos University, Madrid, Spain; 6 Fertility Clinic, Department of Gynaecology, Fertility and Obstetrics, Copenhagen University Hospital – Rigshospitalet, Copenhagen, Denmark; 7 Department of Clinical Medicine, Faculty of Medical and Health Sciences, University of Copenhagen, Copenhagen, Denmark

**Keywords:** anti-Müllerian hormone (AMH), diminished ovarian reserve, follitropin delta, live birth, low ovarian response, ovarian response

## Abstract

**Objectives:**

This study evaluated the efficacy of individualized follitropin delta dosing in patients with potential low response, defined by anti-Müllerian hormone (AMH) level <9 pmol/L, undergoing ovarian stimulation for assisted reproductive technology.

**Methods:**

A pooled analysis including 1844 patients from five randomized controlled trials was undertaken. The trials selected for the analysis used individualized dosing of follitropin delta, based on AMH level and body weight. Patients with potential low response (AMH screening level <9 pmol/L [~1.26 ng/mL]; n=329) were compared to patients with potential normal/high response (AMH level ≥9 pmol/L; n=1515). Patients with AMH <9 pmol/L all received the maximum daily dose of follitropin delta (12 µg).

**Results:**

The mean age was 34.6 years for patients with AMH <9 pmol/L and 32.2 years for patients with AMH ≥9 pmol/L, and the median AMH was 6 pmol/L and 20 pmol/L, respectively. In patients with AMH <9 pmol/L, the mean number of oocytes retrieved (6.3 oocytes) was significantly lower (p<0.0001) than in patients with AMH ≥9 pmol/L (10.8 oocytes), while no significant differences were observed for ongoing pregnancy rate (29.2% versus 31.8% in patients with AMH <9 pmol/L versus ≥9 pmol/L [p=0.32]) or live birth rate (28.6% versus 31.4% [p=0.32]). Seventeen patients (5.2%) with AMH <9 pmol/L and 33 patients (2.2%) with AMH ≥9 pmol/L had cycle cancellations due to poor response. Ovarian hyperstimulation syndrome (OHSS) occurred in 5 patients (1.5%) with AMH <9 pmol/L and 112 patients (7.4%) with AMH ≥9 pmol/L.

**Conclusions:**

Follitropin delta dosing in young patients with moderately reduced ovarian reserve, using 12 µg/day for ovarian stimulation, demonstrated clinically relevant fresh-cycle pregnancy outcomes as compared to patients with normal or high ovarian reserve. This pooled analysis adds evidence for the efficacy of follitropin delta across all ovarian reserve subgroups of the infertile population. The study was limited to fresh cycle outcomes; further studies are warranted focusing on cumulative outcomes and older patient populations with low ovarian reserve.

## Introduction

Low ovarian response to ovarian stimulation constitutes a considerable challenge in assisted reproductive technology (ART) treatment due to suboptimal outcomes in terms of low oocyte yields and reduced success rates. Patients with poor ovarian response constitute 10-30% of the total in vitro fertilization/intracytoplasmic sperm injection (IVF/ICSI) population and are generally represented by patients of advanced age with a reduced ovarian reserve (1, 2). Low response to stimulation has consistently been linked to low live birth and cumulative live birth rates (3–6).

There is no agreed definition of what constitutes a low ovarian response. The European Society for Human Reproduction and Embryology (ESHRE) issued the Bologna criteria in the first attempt to standardize the definition of poor ovarian response (7). The Poseidon group (8) proposed replacing the concept of poor ovarian response with low prognosis (9–12). Both the Bologna and Poseidon criteria take the following factors into account: age, ovarian reserve markers and previous ovarian response.

For patients with no information from previous stimulation cycles available, ovarian reserve markers are universally adopted for the assessment of the ovarian reserve, to identify patients with potential low response. By assessing the ovarian reserve, it is possible to tailor treatment to maximize the ovarian response in patients with diminished ovarian reserve (13, 14). Anti-Müllerian hormone (AMH) is one of the most accurate markers of ovarian reserve and is considered the best single predictor of low ovarian response due to its low intra- and inter-cycle variability (15–17). Both the Bologna and Poseidon criteria use AMH as a marker of ovarian reserve. Although recent reviews have highlighted the multifactorial nature of ovarian response prediction and evaluated additional factors, e.g. dynamic ovarian responsiveness and genetic/environmental modulators of ovarian sensitivity (18), AMH remains a robust predictor of ovarian response.

AMH is part of the dosing algorithm of the recombinant follicle-stimulating hormone (rFSH) follitropin delta. The dose of follitropin delta is individualized based on the patients’ AMH and body weight. The algorithm is particularly useful to guide dosing decisions in patients with no previous information on ovarian response. Clinical trials have demonstrated non-inferiority of individualized follitropin delta compared with conventional follitropin alfa in terms of ongoing pregnancy rate (19, 20), and compared with follitropin beta in terms of the number of oocytes retrieved (21). Results from clinical trials also demonstrated that in the general IVF/ICSI population as well as in potential high responders, individualized dosing of follitropin delta reduces the risk of ovarian hyperstimulation syndrome (OHSS) and/or preventive interventions for OHSS with sustained efficacy compared to conventional follitropin alfa or follitropin beta dosing (20–23). However, the performance of follitropin delta in the specific subpopulation of patients with potential low response has not been evaluated. The aim of this pooled analysis was to evaluate the efficacy and safety of follitropin delta in patients with potential low response, identified exclusively using AMH levels. The study compares the subset of patients with AMH <9 pmol/L (~1.26 ng/mL) to patients with AMH ≥9 pmol/L, among patients who were treated with follitropin delta in their first or second IVF/ICSI cycle as participants in either of five randomized controlled trials (RCTs).

## Materials and methods

### Study design

**Figure 1:**
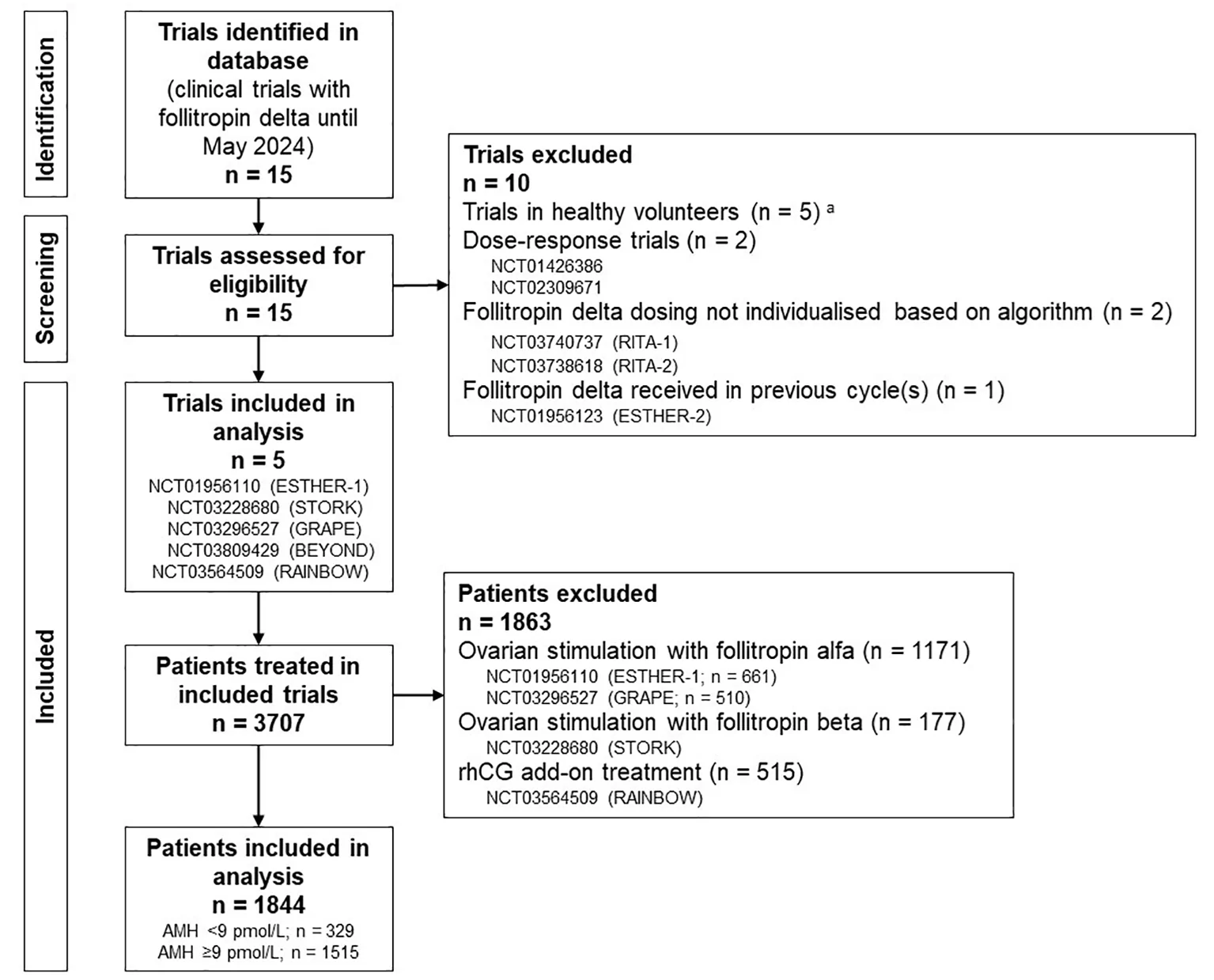
Selection of trials and patients for inclusion in the pooled analysis. AMH, anti-Müllerian hormone; rhCG, recombinant human chorionic gonadotropin ^a^ (34–36).

This was a pooled analysis of five RCTs investigating the clinical outcome of follitropin delta stimulation in patients with potential low response compared to patients with potential normal or high response. The patients included in the analysis had participated in either of five RCTs using individualized dosing of follitropin delta in ovarian stimulation for IVF/ICSI: ESTHER-1 (NCT01956110), STORK (NCT03228680), GRAPE (NCT03296527), RAINBOW (NCT03564509) and BEYOND (NCT03809429). The selected trials were identified from clinical trials on follitropin delta available in the trial database of Ferring Pharmaceuticals up to May 2024. The criteria for trial selection were the inclusion of first-cycle use of follitropin delta for ovarian stimulation and individualized dosing based on the follitropin delta dosing algorithm (**Figure 1**).

The five selected trials were performed in 21 countries in Europe, Asia, North- and South America. The trial protocols and main results were described previously (19–21, 24, 25). All trials had obtained regulatory and ethical approvals and were performed in accordance with the principles of the Declaration of Helsinki, the International Conference on Harmonisation Guidelines for Good Clinical Practice and local regulatory requirements. All patients had provided written informed consent.

### Participants

The five trials included women 18–42 years of age, undergoing their first or second IVF/ICSI cycle (for all patients it was their first ovarian stimulation cycle with follitropin delta and for the vast majority of patients [98.8%] it was their first IVF/ICSI cycle). They had body mass index (BMI) of 17.5–32.0 kg/m^2^ and regular menstrual cycles of 24–35 days. The patients’ infertility diagnoses included tubal infertility, unexplained infertility, endometriosis stage I/II, or partner with male factor infertility. Major exclusion criteria were endometriosis stage III–IV, history of recurrent miscarriage, and use of hormonal preparations during the last menstrual cycle before randomization (thyroid medication was allowed). All patients treated with follitropin delta in the five trials were included in the current pooled analysis. The analysis did not include comparator data due to the use of different or no comparators among the different trials. Patients with potential low response were defined as patients with an AMH level at screening <9 pmol/L. This cut-off was chosen because it approximates the cut-off level used in the Poseidon criteria to define a low ovarian reserve (1.2 ng/mL; corresponding to approximately 8.57 pmol/L (26)). A cut-off of AMH <7 pmol/L (~0.98 ng/mL) was also evaluated in a subgroup analysis. This cut-off was based on the definition of a diminished ovarian reserve. Although there is no universally agreed definition, an AMH cut-off of approximately 1 ng/mL (7.14 pmol/L) is reported to be used in clinical practice (14) and cut-off levels of 1.0, 0.8 and 0.7 ng/mL (7.1, 5.7 and 5.0 pmol/L) have also been reported (13).

### Study procedures

The patients included in the current pooled analysis received follitropin delta (REKOVELLE®, Ferring Pharmaceuticals) for ovarian stimulation in either a gonadotropin-releasing hormone (GnRH) antagonist protocol (83.4% of patients) or a GnRH agonist protocol (16.6% of patients). Follitropin delta was administered subcutaneously at a fixed individualized daily dose, determined by the follitropin delta dosing algorithm, detailed in (19), based on the patient’s serum AMH concentration at screening and body weight at randomization/stimulation Day 1. AMH was assessed centrally using the automated Elecsys AMH immunoassay (Roche Diagnostics International). In the trials performed in Asia (NCT03228680 and NCT03296527), a minimum daily dose of 6 µg was stipulated due to the generally lower body weight in these populations.

For patients undergoing the GnRH antagonist protocol, follitropin delta was initiated on day 2–3 of the menstrual cycle. A GnRH antagonist (0.25 mg/day) was initiated on stimulation day 6 to prevent a premature LH surge and continued throughout the stimulation period. For patients undergoing the agonist protocol, down-regulation started in the mid-luteal phase of the menstrual cycle with a GnRH agonist (0.1 mg/day subcutaneously). If down-regulation was confirmed, follitropin delta was initiated after 14 days and GnRH agonist treatment was continued throughout the stimulation period.

When ≥3 follicles with a diameter ≥17 mm were observed, triggering of final follicular maturation was performed, either with human chorionic gonadotropin (hCG; for patients with <25 follicles ≥12 mm) or with a GnRH agonist (for patients with 25–35 follicles ≥12 mm; not applicable for patients in the GnRH agonist protocol). For patients with 25–35 follicles ≥12 mm, the cycle could also be cancelled, and for all patients with >35 follicles ≥12 mm, the cycle had to be cancelled. If the investigator judged that ≥3 follicles with a diameter ≥17 mm could not be reached by day 20, the cycle was cancelled, or triggering could be performed if 1 or 2 follicles ≥17 mm were observed.

Oocytes were retrieved 36 ± 2 hours after triggering of final follicular maturation. Insemination was performed by IVF or ICSI and embryo development was assessed until the day of transfer. Single or double embryo/blastocyst transfer was performed on Day 3 or Day 5 after hCG administration (for patients who were triggered with a GnRH agonist, all embryos/blastocysts were cryopreserved). In one trial, single blastocyst transfer was mandatory, and in three trials, single blastocyst transfer was mandatory in patients ≤37 years, whereas patients ≥38 years had single blastocyst transfer if a good-quality blastocyst was available, and otherwise double blastocyst transfer. Finally, in one trial, patients <35 years had single embryo transfer if a good-quality embryo was available, otherwise double embryo transfer was performed, whereas patients ≥35 years had double embryo transfer. Vaginal progesterone was provided from oocyte retrieval until pregnancy (positive hCG test, clinical pregnancy or ongoing pregnancy, depending on trial) for luteal phase support. Ongoing pregnancy was confirmed by ultrasound at 10–11 weeks after transfer and all pregnancies were followed until birth.

### Study outcomes

The study outcomes include average daily dose, total gonadotropin dose, duration of stimulation, number of follicles ≥12 mm and ≥17 mm at end of stimulation, cycle cancellation rate (cancellations due to poor ovarian response), number of oocytes retrieved, number of good-quality embryos/blastocysts, and ongoing pregnancy and live birth rates in the fresh cycle. Safety outcomes focus on OHSS rates as this is the most clinically relevant adverse drug reaction observed with ovarian stimulation.

### Statistical analysis

Patients were selected from the trial database according to the criteria illustrated in **Figure 1**. Tabulations are based on all patients exposed to follitropin delta, with patients divided into two groups based on AMH level at screening (<9 pmol/L and ≥9 pmol/L). Patients with a missing ongoing pregnancy assessment were considered not pregnant unless a subsequent assessment confirmed an ongoing pregnancy. Missing values for the number of oocytes retrieved were imputed as zero. If no oocytes were retrieved, the number of good-quality embryos/blastocysts was set to zero. No imputation was performed for other missing data.

Data are presented as mean (± standard deviation), mean [95% CI], median (interquartile range), number (percentage) or percentage [95% CI]. The variables number of oocytes retrieved, ongoing pregnancy rate and live birth rate were compared between the two groups (AMH <9 pmol/L and ≥9 pmol/L) using the Mantel-Haenszel method. These variables were also compared between patients with AMH <7 pmol/L and AMH ≥7 pmol/L. No adjustment for multiple comparisons was performed. In addition, a subgroup analysis of live birth rate by age group (<35, 35-37, and 38–42 years) and AMH group (AMH <9 pmol/L and ≥9 pmol/L) was performed.

## Results

### Selection of trials and patients

At the time of the analysis, the Ferring trial database included 15 trials with follitropin delta, of which five trials were performed in healthy volunteers and did not generate any outcome data, two trials evaluated dose-response, two trials did not use the algorithm for follitropin delta dosing and one trial evaluated follitropin delta in repeated cycles. The remaining five trials were included in the analysis (**Figure 1**). Out of the 3707 patients treated in the trials, 1863 patients were excluded from the analysis due to treatment with a comparator (follitropin alfa or follitropin beta) or the use of hCG as an add-on treatment. All patients exposed to follitropin delta, in total 1844 patients, were included in the analysis (**Figure 1**). Of these, a total of 1558 patients (84.4%) underwent fresh embryo/blastocyst transfer.

### Patient characteristics

Of the 1844 patients in the analysis, 329 patients (17.8%) had AMH <9 pmol/L and 1515 patients (82.2%) had AMH ≥9 pmol/L. The baseline characteristics are shown in **Table 1**. Patients with AMH <9 pmol/L were on average 2.4 years older than patients with AMH ≥9 pmol/L and had an approximately three-fold lower median AMH level. In the AMH <9 pmol/L group, 55% of the patients were ≥35 years (27.4% were 35–38 years and 27.7% were 38–42 years) and in the AMH ≥9 pmol/L group, 31% of the patients were ≥35 years (21.3% were 35–37 years and 10.0% were 38–42years). The distribution of stimulation protocols was the same within both AMH groups (17% of patients treated in a GnRH agonist protocol and 83% in a GnRH antagonist protocol). For 99% of the patients in each AMH group, it was their first IVF/ICSI cycle.

**Table 1:** Demographic and baseline characteristics.

Characteristic	AMH <9 pmol/L (n = 329)	AMH ≥9 pmol/L (n = 1515)
Age (years)	34.6 ± 3.9	32.2 ± 4.0
Age, grouped (years)		
<35	148 (45.0)	1040 (68.6)
35-37	90 (27.4)	323 (21.3)
38-42	91 (27.7)	152 (10.0)
AMH (pmol/L)	6.4 (4.6-7.8)	20.1 (14.4-28.9)
Weight (kg)	64.3 ± 10.3	61.4 ± 11.3
BMI (kg/m^2^)	23.7 ± 3.4	22.8 ± 3.3
Protocol		
Agonist	55 (16.7)	251 (16.6)
Antagonist	274 (83.3)	1264 (83.4)
IVF/ICSI cycle		
First	325 (98.8)	1497 (98.8)
Second	4 (1.2)	18 (1.2)

Values are mean ± standard deviation, median (interquartile range), or number (percentage).

AMH, anti-Müllerian hormone; BMI, body mass index.

### Ovarian response, pregnancy and safety outcomes

Ovarian response, pregnancy outcomes and safety data regarding OHSS are displayed by AMH group in **Table 2**.

**Table 2:** Ovarian response, pregnancies and OHSS.

Characteristic	AMH <9 pmol/L (n = 329)	AMH ≥9 pmol/L (n = 1515)
Ovarian response		
Average daily dose (µg)	12.0 ± 0.1	9.4 ± 2.4
Total dose (µg)	110.6 ± 25.2	86.2 ± 27.1
Duration of gonadotropin treatment (days)	9.2 ± 2.1	9.2 ± 1.9
Cycle cancellations due to poor response	17 (5.2)	33 (2.2)
Follicles ≥12 mm at EOS	7.0 ± 3.4	11.6 ± 5.2
Follicles ≥17 mm at EOS	3.8 ± 1.6	4.8 ± 2.4
Oocytes retrieved^a^	6.4 ± 3.8	10.8 ± 6.0
Good-quality embryos (Day 3)^a,b^	2.7 ± 2.4	4.4 ± 3.6
Good-quality embryos/oocytes retrieved (%)^c^	43.5 ± 29.1	41.7 ± 24.9
Good-quality blastocysts (Day 5)^a,d^	1.4 ± 1.6	2.3 ± 2.5
Good-quality blastocysts/oocytes retrieved (%)^e^	22.6 ± 22.1	21.0 ± 19.0
Pregnancy		
Ongoing pregnancy	96 (29.2)	482 (31.8)
Live birth	94 (28.6)	475 (31.4)
Live birth by age group		
<35 years	53/148 (35.8)	332/1040 (31.9)
35–37 years	25/90 (27.8)	101/323 (31.3)
38–42 years	16/91 (17.6)	42/152 (27.6)
OHSS		
Overall OHSS	5 (1.5)	112 (7.4)
Early OHSS	1 (0.3)	72 (4.8)
Late OHSS	4 (1.2)	40 (2.6)

Values are mean ± standard deviation, percentage, number (percentage), or number of patients with observation/number of patients (percentage).

AMH, anti-Müllerian hormone; EOS, end of stimulation; OHSS, ovarian hyperstimulation syndrome.

^a^ Per started cycle.

^b^ Embryo quality on Day 3 was assessed in all trials and data are based on the full study population.

^c^ Patients with oocytes retrieved; n_(AMH <9 pmol/L)_ = 309, n_(AMH ≥9 pmol/L)_ = 1463.

^d^ Blastocyst quality on Day 5 was assessed in all trials with transfer on Day 5 (ESTHER-1, STORK, BEYOND and RAINBOW) and data are based on 1345 patients; n_(AMH <9 pmol/L)_ = 294, n_(AMH ≥9 pmol/L)_ = 1051.

^e^ Patients with oocytes retrieved in trials with transfer on Day 5; n_(AMH <9 pmol/L)_ = 275, n_(AMH ≥9 pmol/L)_ = 1020.

Following the dosing algorithm for follitropin delta (specifying the maximum daily dose for patients with AMH <15 pmol/L), all patients with AMH <9 pmol/L received a fixed daily dose of 12 µg follitropin delta, resulting in a mean total dose of 110.6 µg, while the mean daily and total follitropin delta dose was 9.4 µg and 86.2 µg in patients with AMH ≥9 pmol/L. The mean duration of stimulation was 9.2 days in both groups. In the AMH <9 pmol/L group, 17 patients (5.2%) had their cycles cancelled due to poor response and in the AMH ≥9 pmol/L group, 33 patients (2.2%) had cycle cancellations. A total of 309 (93.9%) of the patients with AMH <9 pmol/L and 1463 (96.6%) of the patients with AMH ≥9 pmol/L had oocytes retrieved. Embryo/blastocyst transfer was performed in 270 (82.1%) of the patients with AMH <9 pmol/L and 1288 (85.0%) of the patients with AMH ≥9 pmol/L.

Comparisons between AMH groups for number of oocytes retrieved, ongoing pregnancy rate and live birth rate are presented in **Table 3**. The mean number of oocytes retrieved was significantly lower (p<0.0001) in patients with AMH <9 pmol/L (6.3 oocytes) compared to patients with AMH ≥9 pmol/L (10.8 oocytes), while there were no significant differences in ongoing pregnancy rate (29.2% in the AMH <9 pmol/L group and 31.8% in the AMH ≥9 pmol/L group [risk difference -2.9; p=0.32]), or live birth rate (28.6% and 31.4%, respectively [risk difference -2.9; p=0.32]. Similar results were observed when comparing patients with AMH <7 pmol/L to patients with AMH ≥7 pmol/L; the mean number of oocytes were significantly lower (p<0.0001) in patients with AMH <7 pmol/L (5.5 oocytes) compared to patients with AMH ≥7 pmol/L (10.5 oocytes), while the ongoing pregnancy and live birth rates were 26.5% versus 31.9% (risk difference -5.3; p=0.13) and 26.5% versus 31.4% (risk difference -4.5; p=0.19). A comparison of live birth rate by age and AMH showed no significant differences between the AMH <9 pmol/L group and the ≥9 pmol/L group in patients <35 years or 35–37 years, while a significant difference was observed in patients 38–42 years (**Table 4**).

**Table 3:** Treatment outcomes – comparisons between AMH groups.

Outcome	AMH subgroup		Mean/Risk difference (95% CI)	p-value^a^
	AMH <9 pmol/L (n = 329)	AMH ≥9 pmol/L (n = 1515)		
Oocytes retrieved, mean [95% CI]^b^	6.3 [5.7; 7.0]	10.8 [10.5; 11.1]	-4.5 [-5.1; -3.8]	<0.0001
Ongoing pregnancy, % [95% CI]^c^	29.2 [24.3; 34.1]	31.8 [29.5; 34.2]	-2.9 [-8.4; 2.6]	0.3152
Live birth, % [95% CI]^c^	28.6 [23.7; 33.5]	31.4 [29.0; 33.7]	-2.9 [-8.4; 2.6]	0.3156
	AMH <7 pmol/L (n = 200)	AMH ≥7 pmol/L (n = 1644)		
Oocytes retrieved, mean [95% CI]^b^	5.5 [4.7; 6.3]	10.5 [10.3; 10.8]	-5.0 [-5.9; -4.2]	<0.0001
Ongoing pregnancy, % [95% CI]^c^	26.5 [20.4; 32.6]	31.9 [29.7; 34.2]	-5.3 [-11.9; 1.3]	0.1304
Live birth, % [95% CI]^c^	26.5 [20.4; 32.6]	31.4 [29.1; 33.6]	-4.5 [-11.1; 2.0]	0.1935

^a^ Comparing subgroups where p-value is for testing the null hypothesis of ‘equal effect’ against the alternative of ‘different effect’.

^b^ Mean = least squares mean. Pooled analysis is adjusted for the trial.

^c^ Percentage of patients with observation. Pooled analysis done using Mantel-Haenszel method.

AMH, anti-Müllerian hormone; CI, confidence interval.

**Table 4:** Live birth rate by age group – comparisons between AMH groups.

Outcome	AMH subgroup		Mean/Risk difference (95% CI)	p-value^a^
	AMH <9 pmol/L (n = 329)	AMH ≥9 pmol/L (n = 1515)		
Live birth by age group, % [95% CI]^b^				
<35 years	35.8 [28.1; 43.5]	31.9 [29.1; 34.8]	3.3 [-5.1; 11.6]	0.4336
35–37 years	27.8 [18.5; 37.0]	31.3 [26.2; 36.3]	-1.0 [-11.7; 9.7]	0.8579
38–42 years	17.6 [9.8; 25.4]	27.6 [20.5; 34.7]	-11.4 [-22.1; -0.7]	0.0490

^a^ Comparing subgroups where p-value is for testing the null hypothesis of ‘equal effect’ against the alternative of ‘different effect’.

^b^ Percentage of patients with observation. Pooled analysis done using Mantel-Haenszel method.

AMH, anti-Müllerian hormone; CI, confidence interval.

Five cases of OHSS (1.5%) were observed in patients with AMH <9 pmol/L and 112 cases (7.4%) were observed in patients with AMH ≥9 pmol/L.

## Discussion

Patients with low ovarian response constitute a substantial proportion of the IVF/ICSI patient population. A low response to ovarian stimulation is associated with impaired success rates and thereby poses a burden to the patients and presents a challenge for the treating physician.

In the present pooled analysis, covering patients from five RCTs performed in the general IVF/ICSI population, patients with potential low response were identified based on the ovarian reserve marker AMH. In the study population, 17.8% of the patients were potential low responders as defined by an AMH <9 pmol/L. The analysis demonstrated that in this group of patients, follitropin delta dosing (12 µg/day as recommended by the algorithm) produced an appropriate ovarian response and a fresh live birth rate (28.6%) not significantly different from the fresh live birth rate of patients with potential normal and hyperresponse (31.4%). Even though, as expected, the number of oocytes retrieved was significantly lower in patients with AMH <9 pmol/L as compared to patients with AMH ≥9 pmol/L, this did not translate into significantly different fresh cycle pregnancy outcomes. The efficacy of follitropin delta was even evident in the subgroup of patients with a further diminished ovarian reserve (AMH <7 pmol/L), although the fresh live birth rate of 26.5% was numerically lower compared to patients with AMH ≥7 pmol/L (31.4%).

The figures reported are promising compared with previous studies on patients with low response, reporting live birth rates ranging from 7% to 27% (27–29). Furthermore, the cycle cancellation rate due to poor ovarian response was 5.2% in patients with AMH <9 pmol/L, which can be compared with 24% reported by Polyzos et al. (29) and 17% by Leijdekkers et al. (27). However, it should be mentioned that the poor/low responders in the referenced studies are not fully comparable to the potential low responders in the present study in terms of AMH levels and number of previous stimulation cycles.

It should also be noted that the patient population in the present analysis represents patients at the higher end of the spectra of low responders. On average, the patients in the AMH <9 pmol/L group were 2.4 years older compared to patients in the AMH ≥9 pmol/L group. This aligns with expectations of reduced ovarian response along with increased age. Furthermore, the median AMH level of approximately 6 pmol/L in the AMH <9 pmol/L group, compared to 20 pmol/L in the AMH ≥9 pmol/L group, demonstrates this subpopulation’s substantially reduced ovarian reserve. Nevertheless, the patient population in the AMH <9 pmol/L group was relatively young with a mean age of 34.6 years, and considering the AMH cut-off at 9 pmol/L, the median AMH level of 6.4 pmol/L is relatively high compared to the general population of low responders.

The efficacy and safety of individualized follitropin delta were previously demonstrated in the general IVF/ICSI population and in potential high responders (19–25). The current study confirms the adequate performance of 12 µg/day of follitropin delta in the subpopulation of patients with potential low response. Hence, follitropin delta demonstrates consistent efficacy and safety clinical outcomes across the infertile patient subpopulations. The use of follitropin delta in low prognosis IVF patients is also supported by recent retrospective and real-world observational studies (30, 31).

It is well established that live birth rates are influenced by age (27) and reduced rates with increasing age were confirmed also in this study. In the overall analysis, patients in the AMH <9 pmol/L group had a mean age below 35 years, which likely explains the favorable live birth rates observed. Importantly, within this clinically relevant population, individualized dosing with follitropin delta was not associated with any reduction in fresh cycle clinical outcomes. On the contrary, outcomes were comparable between patients with AMH <9 pmol/L and AMH ≥9 pmol/L, supporting the robustness of the dosing algorithm and indicating that it does not underestimate ovarian stimulation requirements in young patients.

In patients undergoing their first stimulation cycle, the follitropin delta-specific dosing algorithm guides dosing by predictions of ovarian response based on the ovarian reserve marker AMH. In the clinical landscape of individualized dosing, follitropin delta provides a balanced and validated dosing algorithm with proven efficacy and with the advantage of a fixed daily dose throughout stimulation.

A limitation of this study is the relatively small sample size of patients with AMH <9 pmol/L, as well as the relatively young study population, limiting generalizability of the findings to older patient populations. Another limitation is that no comparator data are included. Due to differences in comparators used in the trials (follitropin alfa, follitropin beta or none), a consistent evaluation was not possible. Inclusion of comparator data would have strengthened the study findings. A major limitation of the study is that it only covers outcomes from the fresh cycle. With the increasing number of cryopreserved cycles, cumulative live birth rate is the preferred outcome for a complete measure of treatment success (32, 33). Cumulative live birth rates could not be assessed within the scope of this pooled analysis, as outcome data from frozen embryo transfer cycles were not collected in all trials. However, it should be noted that the focus on fresh cycle outcomes is aligned with the clinical rationale for using the follitropin delta dosing algorithm, targeting a range of 8–14 oocytes with the aim to proceed to fresh embryo transfer. Still, future studies including cumulative outcomes, would provide a more comprehensive evaluation. Inclusion of cryopreserved transfer cycles in the analysis would favor the AMH ≥9 pmol/L group, both because patients with a high response and all embryos/blastocyst cryopreserved could have a positive outcome in a subsequent cryopreserved cycle, and also because this group had more excess embryos/blastocysts for cryopreservation. Finally, generalizability of results is limited by the trial eligibility criteria, excluding certain subgroups of patients, e.g. patients with endometriosis stage III–IV.

In conclusion, follitropin delta dosing in patients with potential low ovarian response, using 12 µg/day for ovarian stimulation, appears to yield fresh cycle pregnancy outcomes comparable to those observed in potential normoresponders and hyperresponders. These findings support the consistency of the follitropin delta dosing approach across ovarian reserve subgroups and suggest that treatment efficacy is maintained in this population. This pooled analysis contributes to the growing evidence base supporting the use of follitropin delta in a broad infertile population. Further studies are warranted to confirm these findings, particularly in older patients with low ovarian reserve.

## Data Availability

The data analyzed in this study is subject to the following licenses/restrictions: Data from previously published studies can be found in the original publications. Ferring may provide access to aggregated or de-identified data upon reasonable request, subject to internal review, applicable legal requirements, and a data access agreement. Requests to access these datasets should be directed to Rita.Lobo@ferring.com.

## References

[1] La Marca A Sunkara SK . Individualization of controlled ovarian stimulation in IVF using ovarian reserve markers: from theory to practice. Hum Reprod Update. (2014) 20:124–40. doi: 10.1093/humupd/dmt037 24077980

[2] Oehninger S . Poor responders in *in vitro* fertilization (IVF) therapy: the challenge continues. Facts Views Vis Obgyn. (2011) 3:101–8 24753855 PMC3987493

[3] Devesa M Tur R Rodríguez I Coroleu B Martínez F Polyzos NP . Cumulative live birth rates and number of oocytes retrieved in women of advanced age. A single centre analysis including 4500 women ≥38 years old. Hum Reprod. (2018) 33:2010–7. doi: 10.1093/humrep/dey295 30272168

[4] Drakopoulos P Blockeel C Stoop D Camus M de Vos M Tournaye H . Conventional ovarian stimulation and single embryo transfer for IVF/ICSI. How many oocytes do we need to maximize cumulative live birth rates after utilization of all fresh and frozen embryos? Hum Reprod. (2016) 31:370–6. doi: 10.1093/humrep/dev316 26724797

[5] Neves AR Montoya-Botero P Sachs-Guedj N Polyzos NP . Association between the number of oocytes and cumulative live birth rate: A systematic review. Best Pract Res Clin Obstet Gynaecol. (2023) 87:102307. doi: 10.1016/j.bpobgyn.2022.102307 36707342

[6] Polyzos NP Drakopoulos P Parra J Pellicer A Santos-Ribeiro S Tournaye H . Cumulative live birth rates according to the number of oocytes retrieved after the first ovarian stimulation for in vitro fertilization/intracytoplasmic sperm injection: a multicenter multinational analysis including ∼15,000 women. Fertil Steril. (2018) 110:661–70.e1. doi: 10.1016/j.fertnstert.2018.04.039 30196963

[7] Ferraretti AP La Marca A Fauser BC Tarlatzis B Nargund G Gianaroli L . ESHRE consensus on the definition of 'poor response' to ovarian stimulation for *in vitro* fertilization: the Bologna criteria. Hum Reprod. (2011) 26:1616–24. doi: 10.1093/humrep/der092 21505041

[8] Poseidon Group (Patient-Oriented Strategies Encompassing IndividualizeD Oocyte Number) Alviggi C Andersen CY Buehler K Conforti A De Placido G . A new more detailed stratification of low responders to ovarian stimulation: from a poor ovarian response to a low prognosis concept. Fertil Steril. (2016) 105:1452–3. doi: 10.1016/j.fertnstert.2016.02.005 26921622

[9] Papathanasiou A . Implementing the ESHRE 'poor responder' criteria in research studies: methodological implications. Hum Reprod. (2014) 29:1835–8. doi: 10.1093/humrep/deu135 24916434

[10] Reig A Garcia-Velasco JA Seli E . Bologna vs. POSEIDON criteria as predictors of the likelihood of obtaining at least one euploid embryo in poor ovarian response: an analysis of 6,889 cycles. Fertil Steril. (2023) 120:605–14. doi: 10.1016/j.fertnstert.2023.05.007 37187313

[11] Roque M Haahr T Esteves SC Humaidan P . The POSEIDON stratification - moving from poor ovarian response to low prognosis. JBRA Assist Reprod. (2021) 25:282–92. doi: 10.5935/1518-0557.20200100 33565297 PMC8083858

[12] Sarkar P Romanski PA Devine K . First Bologna, then Poseidon: What are we still missing to personalize care for patients undergoing assisted reproductive technology with a poor prognosis? Fertil Steril. (2023) 120:615–6. doi: 10.1016/j.fertnstert.2023.06.028 37391060

[13] Cohen J Chabbert-Buffet N Darai E . Diminished ovarian reserve, premature ovarian failure, poor ovarian responder--a plea for universal definitions. J Assist Reprod Genet. (2015) 32:1709–12. doi: 10.1007/s10815-015-0595-y 26463876 PMC4681731

[14] Morris ME . Evaluation and management of infertility in females of advancing age, in: Uptodate (2023). Available online at: https://www.wolterskluwer.com/en/solutions/uptodate (Accessed March 19, 2026).

[15] Huang J Lin J Gao H Wang Y Zhu X Lu X . Anti-mullerian hormone for the prediction of ovarian response in progestin-primed ovarian stimulation protocol for IVF. Front Endocrinol (Lausanne). (2019) 10:325. doi: 10.3389/fendo.2019.00325 31191453 PMC6547790

[16] ESHRE Guideline Group on Ovarian Stimulation Ata B Bosch E Broer S Griesinger G Grynberg M . ESHRE guideline: ovarian stimulation for IVF/ICSI: an update in 2025. Hum Reprod. (2026) 24:deag018. doi: 10.1093/humrep/deag018 41732035 PMC13061131

[17] Racca A Dominic S Polyzos NP . Editorial: Diminished ovarian reserve and poor ovarian response: diagnostic and therapeutic management. Front Physiol. (2022) 13:827678. doi: 10.3389/fphys.2022.827678 35928567 PMC9345116

[18] Xu H Feng H Liu X Yin H Wang X Luo Z . Assessment indicators of ovarian response during controlled ovarian stimulation: influencing factors and clinical value. Front Endocrinol (Lausanne). (2026) 17:1793202. doi: 10.3389/fendo.2026.1793202 42181183 PMC13189822

[19] Nyboe Andersen A Nelson SM Fauser BC Garcia-Velasco JA Klein BM Arce J-C . Individualized versus conventional ovarian stimulation for in vitro fertilization: a multicenter, randomized, controlled, assessor-blinded, phase 3 noninferiority trial. Fertil Steril. (2017) 107:387–96 e384. doi: 10.1016/j.fertnstert.2016.10.033 27912901

[20] Qiao J Zhang Y Liang X Ho T Huang HY Kim SH . A randomised controlled trial to clinically validate follitropin delta in its individualised dosing regimen for ovarian stimulation in Asian IVF/ICSI patients. Hum Reprod. (2021) 36:2452–62. doi: 10.1093/humrep/deab155 34179971 PMC8373472

[21] Ishihara O Arce J-C Japanese Follitropin Delta Phase 3 Trial Group . Individualized follitropin delta dosing reduces OHSS risk in Japanese IVF/ICSI patients: a randomized controlled trial. Reprod BioMed Online. (2021) 42:909–18. doi: 10.1016/j.rbmo.2021.01.023 33722477

[22] Fernandez-Sanchez M Visnova H Yuzpe A Klein BM Mannaerts B Arce J-C . Individualization of the starting dose of follitropin delta reduces the overall OHSS risk and/or the need for additional preventive interventions: cumulative data over three stimulation cycles. Reprod BioMed Online. (2019) 38:528–37. doi: 10.1016/j.rbmo.2018.12.032 30713022

[23] Visnova H Papaleo E Martin FS Koziol K Klein BM Mannaerts B . Clinical outcomes of potential high responders after individualized FSH dosing based on anti-Mullerian hormone and body weight. Reprod BioMed Online. (2021) 43:1019–26. doi: 10.1016/j.rbmo.2021.08.024 34756645

[24] Fernandez-Sanchez M Visnova H Larsson P Yding Andersen C Filicori M Blockeel C . A randomized, controlled, first-in-patient trial of choriogonadotropin beta added to follitropin delta in women undergoing ovarian stimulation in a long GnRH agonist protocol. Hum Reprod. (2022) 37:1161–74. doi: 10.1093/humrep/deac061 35451013 PMC9156848

[25] Lobo R Soerdal T Ekerhovd E Cohlen B Porcu E Schenk M . BEYOND: a randomized controlled trial comparing efficacy and safety of individualized follitropin delta dosing in a GnRH agonist versus antagonist protocol during the first ovarian stimulation cycle. Hum Reprod. (2024) 39:1481–94. doi: 10.1093/humrep/deae092 38723189 PMC11759129

[26] Rekovelle, SmPC . Available online at: https://www.ema.europa.eu/en/documents/product-information/rekovelle-epar-product-information_en.pdf (Accessed March 19, 2026).

[27] Leijdekkers JA Eijkemans MJC van Tilborg TC Oudshoorn SC van Golde RJT Hoek A . Cumulative live birth rates in low-prognosis women. Hum Reprod. (2019) 34:1030–41. doi: 10.1093/humrep/dez051 31125412 PMC6555622

[28] Mashayekhi M Barabi F Arabipoor A Zolfaghari Z . Live birth rates in different subgroups of poor ovarian responders according to Bologna and POSEIDON group classification criteria. J Gynecol Obstet Hum Reprod. (2021) 50:102169. doi: 10.1016/j.jogoh.2021.102169 34044136

[29] Polyzos NP Nwoye M Corona R Blockeel C Stoop D Haentjens P . Live birth rates in Bologna poor responders treated with ovarian stimulation for IVF/ICSI. Reprod BioMed Online. (2014) 28:469–74. doi: 10.1016/j.rbmo.2013.11.010 24581984

[30] Thi HD Nguyen DK Thanh TN Ngoc NN Pham HTM Van TT . Evaluation of the follitropin delta and clomiphene citrate combination in low prognosis women undergoing *in vitro* fertilization: A retrospective study. Clin Exp Obstet Gynecol. (2025) 52:43781. doi: 10.31083/CEOG43781

[31] Deloire AC Porcu-Buisson G Lefebvre R Bernot M . A multicentric real-world observational study to describe the use and efficacy of follitropin delta for IVF/ICSI procedures in patients at risk of hypo-response. Front Reprod Health. (2025) 7:1650946. doi: 10.3389/frph.2025.1650946 41000267 PMC12457373

[32] Saket Z Kallen K Lundin K Magnusson A Bergh C . Cumulative live birth rate after IVF: trend over time and the impact of blastocyst culture and vitrification. Hum Reprod Open. (2021) 2021:hoab021. doi: 10.1093/hropen/hoab021 34195386 PMC8240131

[33] Maheshrawi A McLernon D Bhattacharaya S . Cumulative live birth rate: time for a consensus? Hum Reprod. (2015) 30:2703–7. doi: 10.1093/humrep/dev263 26466912

[34] Olsson H Sandström R Grundemar L . Pharmacokinetic and pharmacodynamic properties of recombinant follicle-stimulating hormone (rFSH) derived from a human cell line compared with rFSH from a non-human cell line. J Clin Pharmacol. (2014) 54:1299–307. doi: 10.1002/jcph.328 24800998

[35] Olsson H Sandström R Bagger Y . Dose-exposure proportionality of a novel recombinant follicle-stimulating hormone (rFSH), FE 999049, derived from a human cell line, with comparison between Caucasian and Japanese women after subcutaneous administration. Clin Drug Investig. (2015) 35:247–53. doi: 10.1007/s40261-015-0276-8 25773354 PMC4368841

[36] Shao F Jiang Y Ding S Larsson P Pinton P Jonker DM . Pharmacokinetics and safety of follitropin delta in gonadotropin down-regulated healthy Chinese women. Clin Drug Investig. (2023) 43:37–44. doi: 10.1007/s40261-022-01232-9 36478528 PMC9834375

